# 

**Published:** 1907-04

**Authors:** 


### THE GEORGIA MEDICAL ASSOCIATION.

The Fifty-Eighth annual meeting of the Medical Association of Georgia will be held in the De Soto Hotel at Savannah on April 17, 18, 19.

The program has been divided into medical and surgical sections which will facilitate the reading of papers and permit the members to attend the section in which they are most interested. The program is large and varied and the topics are of more than usual interest.

There will be a subscription banquet at the Casino, Thunderbolt on Thursday and an oyster roast at Tybee Island on Friday. The privileges of the Savannah Clubs have been accorded the members and as it is characteristic of that city nothing will be omitted that hospitality can suggest to make the visiting members have an enjoyable time. The attendance will probably be large on account of the great increase in membership and the attractive features, literary and social offered by the meeting. The contingent from Atlanta will be ample.

The following is the official program:

### WEDNESDAY, APRIL 17

#### MORNING SESSION
9 TO 10 O'CLOCK

Prayer—By the Rev. Wm. F. Pickard.
Address of welcome on behalf of the city---Mayor George W. Tiedeman.
Address of welcome in behalf of the local profession—E. R. Corson, M.D.
Response to address of welcome—Thos. D. Coleman, M.D.
Report of House of Delegates.
Report of Committee on Arrangement.
Report of Committee on Scientific Work.

#### 10 TO 1 O'CLOCK—READING OF PAPERS.

#### SECTION OF GENERAL MEDICINE.

1. Entero-Colitis in Children—Thos. J. McArthur, M. D., Cordele.
2. Entero-Colitis—H. H. McGee, . D., Savannah.
3. The Training of Epileptic and Feeble-minded Children—Wesley Taylor, M. D., Atlanta.
4. Diphtheria and its Treatment—Robert V. Martin, M. D., Savannah.

5. The Soda Fountain and its Attendant Evils—J. A. Combs, M. D., Locust Grove.
6. Pneumonia—J. T. Longino, M. D., Fairburn.
7. Broncho-Pneumonia—James H. Crawford, M. D., Martin.
8. Tetanus—J. A. Crowther, M. D., Savannah.
9. Report of a case of Angina—T. E. Oertel, M. D., Augusta.

---

10 to 1 O'Clock.

SECTION ON SURGERY.

1. Surgical Shock and its Trea'ment—T. J. Charlton, M. D., Savannah.
2. Bladder Drainage in Old Men—W. S. Goldsmith, M. D., Atlanta.
3. Apparatus for the Treatment of Fractured Femur—Jabez Jones, M. D., Savannah.
4. Diagnosis of Diseases of the Prostate—E. G. Ballenger, M D., Atlanta.
5. Suprabubic Prostatectomy—Eugene R. Corson, M. D., Savannah.
6. Acute Prostatitis—W. L. Champion, M D., Atlanta.
7. Diseases of the Rectum—W. H. Campbell, M. D., Chipley.
8. Irritable and Hypertrophied Sphincter Ani; Causes and Treatment—J. L. Farmer, M. D., Savannah.

---

### AFTERNOON SESSION.

3 to 6 O'Clock.

---

SECTION ON MEDICINE.

10. My Experience with Continued Fevers—J. W. Palmer, M. D., Ailey.
11. Report of Fifty Dwellings Infected with Typhoid Fever—W. P. Williams, M. D., Blackshear.
12. Treatment of Typhoid Fever—A. R. Scott, M. D., McDonough.
13. The Treatment of Typhoid Fever—J. B. Warnell, M. D., Cairo.
1ϸ. Paronoia—W. Herbert Adams, M. D., Savannah.
15, Irren—St. J. B. Graham, M. D., Savannah.
16. Infant Feeding—S. A. Visanska, M. D., Atlanta.
17. The Negro Question from a Medical Standpoint—Pryor W. Fitts, M. D., Greenville.
18. Benefits to be deriAed from Our County Medical Societies—J. L. Elrod, M. D., Forsyth.
19. Some Cases of Abdominal Pains—L. C. Roughlin, M. D., Atlanta.
20. The Cataphoric Treatment of Cancer—J. R. Sewell, M. D., Carrollton.

WEDNESDAY NIGHT.

Illustrated Lecture—Tuberculosis—T. E. Oertel, M. D.

---

SECTION ON SURGERY.

9. Reports of Operations for Multiple Abscess of Pelvis with Slough of Six Inches of Colon; Subsequent Anastamosis with Connell Suture; Recovery—E. C. Davis, M. D., Atlanta.

10. Rupture of Uterus; Escape of Fetus into Abdominal Cavity; Operation and Recovery—A. P. Hanie, M. D., Hartwell.
11- A Plea for Careful Surgical Technique. Report of Case of Ante-Partum Hemorrhage Due to Partial Separation of Normally Attached Placenta—Whatley W. Battey, M. D., Augusta.
12. Matas's Operation for Aneurism; Report of Four Cases—Craig Barrow, M. D., Savannah.
13- Face and Neck Operations for Cancer—M. B. Hutchins, M. D., Atlanta.
14. Surgical Anesthesia; Methods and Complications—J. M Sigman, Savannah.
15. Report of Two Cases of Empyema Treated by Aspiration and Irrigation—Chas. Pelham Ward, M. D., Atlanta.

### THURSDAY, APRIL 18.

### MORNING SESSION.

9 to 10 O'Clock.

GENERAL SESSION.

President's Address.

Report of Secretary.

Report of House Delegates.

Report of Committees.

SECTION OF MEDICINE-

10 to 1 O'Clock,

19. Tropical Aphtha or Sprue in Georgia—H. F, Harris, M. D., Atlanta.
20. Personal Results in the Treatment of Tuberculosis—John P. Atkinson, M. D., Milledgeville.
21. Cough and its Treatment in Consumption—M. M. Saliba, M. D., Savannah.
22. Tuberculosis of the Bones, with Report and Presentation of Case—J. L. Jackson, M. D., Savannah.
23. Prevention and Cure of Tuberculosis—J. W. Starr, M. D., Canon.
24. Home Treatment of Tuberculosis—J. E. Sommerfield, M. D., Atlanta.
25. Auto-Intoxication; Its Relation to Neurasthenia—B. P. Oliveros, M. D., Savannah.
26. State Provision for Alcoholic and Drug Habitues—C. C. Stockard, M. D., Atlanta.
27. Obstetrics—J. C. Raley, M. D., North Augusta.
28. Puerperal Eclampsia—Mark H. O'Daniel, M. D,, Jeffersonville.

SECTION ON SURGERY.

10 to 1 O'Clock.

16. Cystocele—George R. White, M. D., Savannah.
17. Nephroptosis—R. R. Kime, Atlanta.
18. A Further Report on the Influence of the Respiration Upon the Development of the Chest Deformity in Scoliosis, with its Relation to the Application of Plaster Jackets—Michael Hoke, M. D , Atlanta.
19. Superficial Inguinal Hernia—Edward G. Jones, M. D., Atlanta.
20. Fractures of the Clavicle and their Treatment—W. F. Westmoreland, M. D., Atlanta.
21. A Case Extraordinary of Laceration of the Perineum—J. G. Earnest, M. D,, Atlanta.
21. Repair of Injuries Due to Parturition—T. P. Waring, M. D., Savannah.
23. Should the Appendix be Removed in All Cases where the Abdomen is Opened for Other Causes?—L. C. Fischer, M. D., Atlanta.

---

AFTERNOON SESSION.

3 to 6 O'Clock.

SCIENTIFIC EXHIBIT.

Thursday afternoon will be devoted to a Scientific Exhibit. Members are earnestly requested to bring or send any pathological specimens of interest, new appliances or apparatus, instruments, etc. These will be shown and explained by the members and will constitute a profitable feature of the meeting.

---

### FRIDAY, APRIL 19.

NORNING SESSION.

9 to 10 O'Clock.

GENERAL SESSION.

Report of House Delegates.

Report of Special Committees.

Miscellaneous Business.

---

SECTION ON MEDICINE.

10 to 1 O'Clock.

29. Methylene Blue in the Treatment of Cancer—H. R. Slack, M. D., LaGrange.
40. Hyperchlorhydria—T. T. Rogers, M. D., Savannah.
31. Preliminary Report on the Relation of Albuminous Putrifaction in the Intestines to Arthritis Deformans (Rheumatoid Arthritis, Osteo-Arthritis):

Its Influence Upon Treatment—C. R. Andrews, M. D. and Michael Hoke, M. D., Atlanta.

32. Why Women are Not More Frequently in as Good Health After Labor as Before Pregnancy—Edward P. Rice, M. D., Augusta.
33. The Germ which Produces Inflammation of the Middle Ear in Atlanta—Claude A. Smith, M. D., Atlanta.
34. Phototherapy, a Potent Therapeutic Agent in the Treatment of Certain Forms of Nervous and Mental Diseases—J. Cheston King, M. D., Atlanta
35. Remarks on some the Ocular Manifestations of Hysteria—W. C. Kellogg, Augusta.
36. The Medical Profession and the Great Social Evil—Archibald Smith, M. D , Atlanta.
37. Blood Pressure in Health and Disease—Ralston Latimore, M. D., Savannah.
38. The Influence of Kræpelin on Modern Psychistry—Martin Cooley, M.D., Savannah.
39. Acne and Rosacea—G O. Brinkley, M. D., Savannah.
40. Opsonins—Lawrence Lee, M. D., Savannah.
4i. The Physician, His Obligations and Duties—Robert B. Barron, M. D., Macon.
42, Report of a Case of Sprue—A. G. Fort, M. D., Lumpkin.

---

### SECTION ON SURGERY.

10 to 1 O'Clock

24. Conservatism in Gynecological Surgery..George A. Wilcox, M. D., Augusta.
25. Discussion of Diseases of the Accessory Nasal Sinues; Description of Operative Procedure on the Parts, Especially of Killian's; Report of Case Done in Georgia—F. E. Cunningham, Macon.
26. Significance of Blood Examinations in Surgical Diagnosis and Prognosis, Willis Jones, M. D., Atlanta.
27. Some Gynecological Cases—W. S. Elkin, M. D., Atlanta.
28. Injuries to the Kidneys—W. B. Crawford, M. D., Savannah.
29. Infusion: Its Indication and Technique—W. Monroe Smith, M. D., Atlanta.
30. Refraction—R, B. Ridley, M. D., Atlanta.
31. Tonsillotomy—H. H. Martin, M. D., Savannah.

---

The Supreme Court of Massachusetts has decided recently that the school authorities have a right to exclude from school unvaccinated children even when a physician's certificate of unfitness for vaccination is presented, if there is an epidemic of smallpox in the district.

